# Atrial Fibrillation Begets Atrial Fibrillation in Small Animals: Characterization of New Rat Model of Spontaneous Atrial Fibrillation

**DOI:** 10.3390/biomedicines13030704

**Published:** 2025-03-13

**Authors:** Alkora Ioana Balan, Vasile Bogdan Halaţiu, Dan Alexandru Cozac, Emilian Comșulea, Cosmin Constantin Mutu, Ioana Aspru, Delia Păcurar, Claudia Bănescu, Marcel Perian, Alina Scridon

**Affiliations:** 1 Physiology Department, University of Medicine, Pharmacy, Science and Technology “George Emil Palade”, 540139 Târgu Mureș, Romaniadeliapacurar99@gmail.com (D.P.);; 2 Cardiology Department, Emergency Institute for Cardiovascular Diseases and Transplantation, 540139 Târgu Mureș, Romania; 3 Center for Advanced Medical and Pharmaceutical Research, University of Medicine, Pharmacy, Science and Technology “George Emil Palade”, 540139 Târgu Mureș, Romania; 4 Doctoral School, University of Medicine, Pharmacy, Science and Technology “George Emil Palade”, 540139 Târgu Mureș, Romania; 5 Emergency Clinical County Hospital, 540139 Târgu Mureș, Romania; 6 Genetics Department, University of Medicine, Pharmacy, Science and Technology “George Emil Palade”, 540139 Târgu Mureș, Romania

**Keywords:** atrial fibrillation, atrial pacing, heart rate variability, molecular remodeling, rat model

## Abstract

**Background/Objectives**: We previously described a rat model of AF induced by long-term transesophageal atrial burst pacing. Here, we further characterize this model by exploring arrhythmia inducibility, spontaneous AF occurrence, and related autonomic and molecular changes. **Methods**: Twelve adult male Wistar rats were randomized into two groups: control (n = 5) and AF (n = 7). The rats in the AF group underwent 10 days of transesophageal atrial pacing. In the control rats, the same protocol was mimicked. Spontaneous AF occurrence and heart rate variability (HRV) were evaluated before, during, and after stimulation. Left atrial RNA levels of *Hcn1*, *Hcn2*, *Hcn4*, and *Pitx2* were evaluated. **Results**: In AF, no animal presented spontaneous AF before stimulation. After stimulation initiation, all AF rats presented spontaneous AF (*p* = 0.08). In the AF rats, HRV analysis revealed a progressive increase in the standard deviation of the RR intervals after atrial stimulation initiation (*p* < 0.01). The left atrial RNA levels of *Hcn4* were higher (*p* = 0.03) and Pitx2 levels were lower (*p* = 0.02) in the AF rats compared to the control group. **Conclusions**: This study validates our previous data and confirms the occurrence of spontaneous AF following long-term atrial pacing in rats. Relatively increased parasympathetic modulation and changes in the atrial expression of *Hcn4*, encoding for *I_f_*, and Pitx2 likely play critical mechanistic roles in this model.

## 1. Introduction

Atrial fibrillation (AF) is the most prevalent sustained cardiac rhythm disorder [1]. The mechanisms involved in AF occurrence have not been completely elucidated and the therapeutic options that are currently available have limited efficacy and are accompanied by non-negligible side effects [1,2].

Animal research has had a fundamental role in the numerous scientific and medical advances made over the last century and continues to contribute to our understanding of pathophysiological phenomena and the identification of new therapeutic targets [3,4]. As AF does not occur “naturally” in most species, experimental models based on electrical stimulation or pharmacological challenge have been developed to provide a working tool in the study of AF [3,4]. Although studies on large animals are highly important for understanding the AF mechanisms, they have several disadvantages, related in particular to the difficulties and costs associated with animal housing and care, as well as to their long life span [3,4].

For a long time, it was considered that reentrant arrhythmias, including AF, are impossible to maintain in small atria [5]. However, several rat models of AF have been developed over the years [3,4]. In most of those studies, AF was induced by electrical stimulation in the presence of favorable conditions such as cholinergic stimulation or asphyxia, settings that can induce unwanted interactions and can interfere with the interpretation of the results [6,7]. Moreover, all previous studies focused on AF inducibility after a single episode of electrical stimulation; the impact of long-term atrial stimulation on spontaneous AF occurrence after electrical stimulation has not been evaluated in small animals. A long-term atrial pacing-induced AF model in rats would allow assessment of AF-related structural, electrical, autonomic, and molecular remodeling and could be used to test both the acute and chronic effects of antiarrhythmic strategies on atrial remodeling and AF. In a previous study, we showed the effectiveness of a long-term atrial electrical stimulation protocol in inducing spontaneous AF in small rodents, demonstrating that the concept of “AF begets AF” also applies to rats [8].

In this study, we aimed to validate this new model of AF induced by long-term transesophageal atrial pacing and to gain mechanistic insights into this model, with a focus on autonomic modulation and atrial transcriptomic changes in the genes related to AF.

## 2. Materials and Methods

### 2.1. Studied Animals

The study was performed on 12 adult male Wistar rats (250–300 g) obtained from the local Experimental Animal Center. All rats underwent a baseline health assessment, and animals that presented any signs of systemic illness, congenital abnormalities, or spontaneous arrhythmic events before the start of the experiment were not included in the study. The animals were randomized into two groups: control (n = 5) and AF (n = 7).

The sample size was chosen in accordance with the general requirements for animal experimentation, to ensure sufficient statistical power to detect meaningful differences between the control and AF groups. The sample size was based on preliminary data obtained from a pilot study that examined the effects of transesophageal atrial electrical stimulation on arrhythmia inducibility and spontaneous AF occurrence. These pilot data were used to estimate the variance and effect size for key outcomes, such as the inducibility of AF and spontaneous AF occurrence. Sample size calculation was performed for each group independently, ensuring that each group was adequately powered to detect differences within and between groups with a statistical power of 80% and an alpha level of 0.05. A slightly larger AF group was chosen because of the invasive nature of the protocol. All rats were housed individually in polycarbonate cages, in a climate-controlled room (21 °C to 23 °C), with a 12 h light/12 h dark cycle and had free access to food and water throughout the study. Experimental protocols were approved by the local Ethics Committee and by the National Sanitary Veterinary and Food Safety Authority (118/27.06.2018) and complied with the International Council for Laboratory Animal Science guidelines (Directive 2010/63/EU).

### 2.2. Transesophageal Programmed Atrial Electrical Pacing and Assessment of Atrial Fibrillation Inducibility

**Figure 1:**
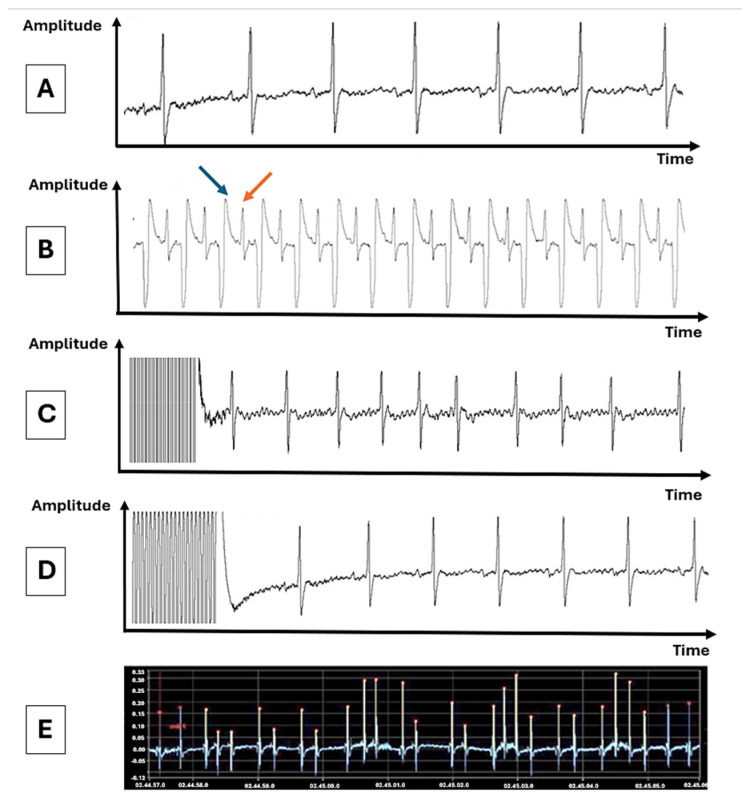
Surface ECG tracings during the transesophageal atrial electrical stimulation protocol (**A**–**D**) and during the continuous ECG monitoring (**E**). (**A**) Baseline surface ECG tracing recorded before application of electrical stimuli. (**B**) ECG tracing demonstrating the correct positioning of the pacing catheter (a narrow QRS complex [orange arrow] is observed after each generated stimulus [blue arrow]). Confirmation of the correct positioning of the catheter during the protocol based on the appearance of (**C**) an atrial fibrillation episode or (**D**) a longer time interval at the end of stimulation, corresponding to the sinus node recovery time. (**E**) ECG tracing recorded during continuous ECG monitoring illustrating an episode of atrial fibrillation. Atrial fibrillation beats are depicted in yellow. Note the complete absence of P waves, the narrow QRS complexes, and the highly irregular ventricular rhythm.

Rats in the AF group underwent a long-term (10-day) transesophageal programmed atrial electrical pacing protocol. Control rats were subjected to the same protocol, but without applying the electrical stimuli. Rats were anesthetized with ketamine/medetomidine mixture (75.0/0.5 mg/kg), and surface electrocardiogram was recorded throughout the protocol (Figure 1A). Atrial electrical stimulation was performed with a transesophageal quadripolar catheter connected to an external pacemaker, as described previously [9. The correct position of the catheter was confirmed by the presence of a narrow QRS complex after each stimulus, generated at a rate of 400 stimuli per minute (Figure 1B). In each animal, 15 successive stimulation cycles of 20 s each, with a free interval of 5 min between cycles, were applied daily for 10 days. To induce AF, the stimuli were applied at a frequency of 4000 stimuli/minute (stimulus duration 6 ms), at a voltage 3 V above the diastolic threshold. The efficiency of the stimulation was confirmed by the appearance, at the end of the stimulation cycle, of AF, defined as a rapid, irregular rhythm, with at least three narrow QRS complexes, and absent P waves (Figure 1C) or of a free interval corresponding to the sinus node recovery time (Figure 1D). At the end of the protocol, the effects of medetomidine were antagonized with atipamezole (1 mg/kg). Baseline heart rate, AF inducibility (expressed as the percentage of stimulation cycles followed by AF episodes), and the average duration of AF episodes were evaluated and compared between the two groups.

### 2.3. Implantation of ECG Radiotelemetry Devices and Spontaneous Arrhythmic Burden Assessment

Each animal was implanted at the beginning of the study with an ECG radiotelemetry device (TA11 CA-F40; Data Sciences International, St. Paul, MN, USA) under isoflurane inhalation anesthesia. The radiofrequency transmitter was placed in a subcutaneous pocket in the dorsolateral part of the abdomen. To obtain a lead II configuration, the ECG electrodes were tunneled and fixed subcutaneously in the right subclavicular region and at the cardiac apex.

**Figure 2:**
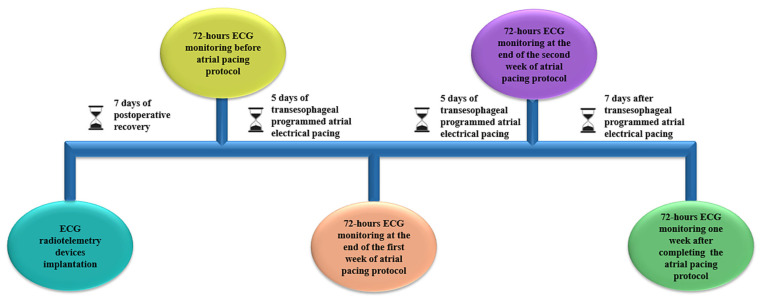
Overview of the experimental protocol with the 4 key ECG monitoring periods.

Continuous ECG monitoring was performed for 72 h/week before, during (at the end of the first and the second weeks of stimulation), and one week after completing the atrial-pacing protocol (Figure 2).

The analysis of ECG recordings was performed with software developed in LabVIEW 2010 (National Instruments, Austin, TX, USA). For each ECG, mean heart rate over 24 h and atrial arrhythmic events (type, number, and duration) were evaluated. The main arrhythmic events monitored were AF (Figure 1E) and atrial premature beats (APBs). The number and duration of atrial arrhythmic events were reported over 24 h intervals and compared between the two groups. In agreement with previous studies [8,9], AF was defined as a rapid, irregular rhythm, with at least three consecutive narrow QRS complexes, and absent P waves. The duration of an AF episode was measured from the first arrhythmic beat to the first subsequent sinus beat. Premature atrial depolarizations with different morphology compared to sinus rhythm P waves were defined as APBs.

### 2.4. Heart Rate Variability Analysis

Cardiac sympathetic and parasympathetic modulation and sympatho-vagal balance were evaluated by heart rate variability (HRV) analysis based on the continuous ECG recordings obtained before, during, and after the atrial-pacing protocol. For each animal, the variations between consecutive RR intervals in the frequency and temporal domains were analyzed using a program developed in our laboratory using LabVIEW 2010 software (National Instruments, Austin, TX, USA), as described previously [10]. To prevent distortion of the data, all ECG tracings were visually assessed by two independent cardiologists who identified and excluded arrhythmic events and artifacts from the HRV analysis. In the temporal domain, the standard deviation of normal RR intervals (SDNN), root mean square of successive RR-interval differences (RMSSD), and the proportion of adjacent RR intervals that differed by >5 ms (pNN5) were analyzed. In the frequency domain, the low-frequency (LF; 0.3–0.6 Hz) and the high-frequency (HF; 0.6–2.5 Hz) components of the HRV spectrum and the LF/HF ratio were analyzed.

### 2.5. Left Atrial Expression of Atrial Fibrillation-Related Genes

At the end of the ECG monitoring period, the animals were euthanized using an intraperitoneal injection of a terminal dose of sodium pentobarbital (>100 mg/kg). The hearts were explanted, and the left atrium was collected in RNA stabilization solution (RNAlater; Thermo Fisher Scientific, Waltham, MA, USA) for transcriptome analysis.

iPrep PureLink Total RNA Kits and the iPrep Purification Instrument (Thermo Fisher Scientific, Waltham, MA, USA) were used for RNA isolation. Reverse transcription was performed using the SuperScript VILO cDNA Synthesis Kit (Thermo Fisher Scientific, Waltham, MA, USA). To analyze RNA expression levels of the target genes, a customized fast 96-well plate containing TaqMan Gene Expression Assays for the tested genes (Thermo Fisher Scientific, Waltham, MA, USA) was used, as described before [11]. The RNA expression levels of hyperpolarization-activated cyclic nucleotide-gated channels (*Hcn*) 1, 2, and 4, of paired-like homeodomain transcription factor 2 (*Pitx2*), and of one control gene, (i.e., glyceraldehyde 3-phosphate dehydrogenase [GAPDH]) were analyzed. The expression level of the target genes was normalized with GAPDH housekeeping gene levels and compared between the AF and control groups.

### 2.6. Statistical Analysis

Statistical analysis was performed using GraphPad Prism version 10 (GraphPad Software, San Diego, CA, USA). All data were tested for normality and are expressed as means ± standard error of the mean or median and interquartile range, as appropriate. Depending on the distribution of the data, differences between groups were assessed using the Welch corrected unpaired *t*-test or the Mann–Whitney U test. Repeated measures ANOVA or Friedman tests were used to analyze differences within the same group. A *p*-value < 0.05 was considered statistically significant.

## 3. Results

### 3.1. Inducibility of Atrial Fibrillation by Transesophageal Atrial Pacing

No arrhythmic episodes were induced in the control rats. In the stimulated rats, AF was induced in six out of the seven animals (85.7%), with an average global inducibility of 44.0% ± 6.3%. The average duration of the stimulation-induced AF episodes was 123.2 ± 36.4 s.

### 3.2. Spontaneous Arrhythmic Burden in Control and Stimulated Rats

The mean 24 h heart rate was not significantly different between the AF and control groups in any of the continuous ECG monitoring moments (all *p* > 0.05; Table 1).

**Figure 3:**
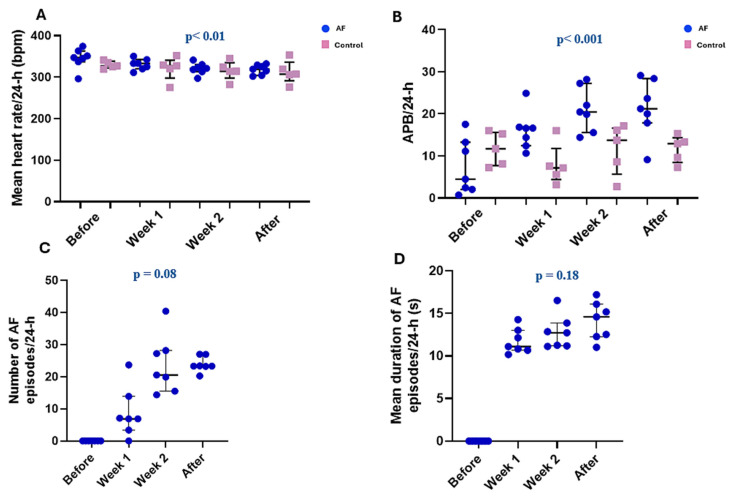
Mean heart rate and number and duration of atrial arrhythmic events during the four continuous ECG monitoring periods. (**A**) Mean heart rate/24 h in the stimulated (AF) and non-stimulated (control) rats. (**B**) Number of atrial premature beats (APBs)/24 h in the stimulated (AF) and non-stimulated (control) rats. (**C**) Number of atrial fibrillation (AF) episodes/24 h in the electrically stimulated rats. (**D**) Average duration of AF episodes/24 h in the electrically stimulated rats. Before—before atrial pacing; week 1—at the end of the first week of atrial pacing; week 2—at the end of the second week of atrial pacing; after—one week after the completion of the atrial-pacing protocol. Data are expressed as individual data points, medians and interquartile range. *p*-values are provided for the AF group and were obtained using the repeated measures ANOVA or Friedman test, as appropriate.

However, in the AF rats, there was a progressive decrease in mean 24 h heart rate (*p* < 0.01), whereas in the control rats, the heart rate remained constant (*p* = 0.52) during the study (Figure 3A).

Before transesophageal electrical stimulation, the number of APBs/24 h was not significantly different between the two groups (*p* = 0.19). However, the number of APBs was significantly higher in the AF rats compared to the control rats after both the first (*p* = 0.01) and the second (*p* = 0.02) weeks of stimulation, as well as after completing the transesophageal electrical stimulation protocol (*p* = 0.01). In the stimulated rats, the number of APBs increased progressively after the initiation of the atrial electrical stimulation protocol (*p* < 0.001; Figure 3B).

In the control group, spontaneous AF episodes were not recorded in any of the periods of continuous ECG monitoring. Similarly, none of the AF animals presented spontaneous AF episodes before transesophageal electrical stimulation. Meanwhile, after the first week of stimulation, six out of the seven (85.7%) AF rats presented spontaneous AF, while after the second week, as well as one week after the end of the stimulation protocol, all AF rats presented episodes of spontaneous AF. In the stimulated rats, the number of AF episodes tended to increase progressively after the initiation of the atrial electrical stimulation protocol (*p* = 0.08; Figure 3C). Meanwhile, the duration of the spontaneous AF episodes remained relatively constant after the initiation of the electrical stimulation protocol (*p* = 0.18; Figure 3D).

### 3.3. Time and Frequency Domain Analysis of Heart Rate Variability

In all four ECG monitoring periods, SDNN, RMSSD, and pNN5 were all similar in the AF and control groups (all *p* > 0.05). Frequency domain parameters (i.e., LF, HF, and LF/HF) were also similar between the two groups (all *p* > 0.05; Table 2).

**Figure 4:**
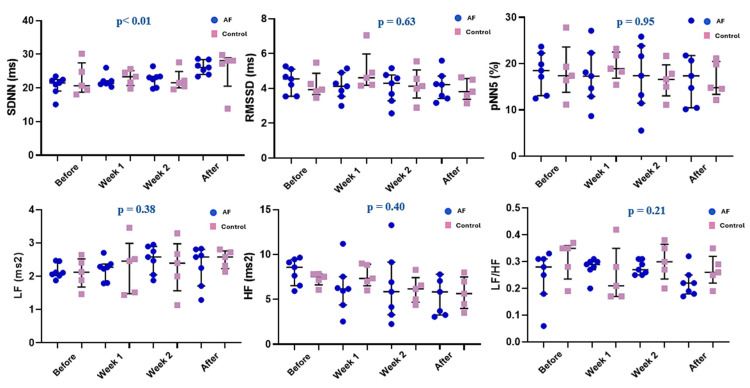
Heart rate variability parameters in the electrically stimulated (AF) and non-stimulated (control) rats during the four periods of continuous ECG monitoring. Before—before atrial pacing; week 1—at the end of the first week of atrial pacing; week 2—at the end of the second week of atrial pacing; after—one week after the completion of the atrial-pacing protocol. HF—high-frequency (0.6–2.5 Hz) components of the heart rate variability spectrum; LF—low-frequency (0.3–0.6 Hz) components of the heart rate variability spectrum; pNN5—proportion of adjacent RR intervals that differed by >5 ms; RMSSD—root mean square of successive RR-interval differences; SDNN—standard deviation of normal RR intervals. Data are expressed as individual data points, medians and interquartile range. *p*-values are provided for the AF group and were obtained using the repeated measures ANOVA or Friedman test, as appropriate.

However, in line with the progressive decrease in mean 24 h heart rate observed in the AF group, SDNN showed a progressive increase after the initiation of the atrial electrical stimulation protocol in this group (*p* < 0.01). All other analyzed parameters (i.e., RMSSD, pNN5, LF, HF, and LF/HF) remained constant in this group throughout the study (all *p* > 0.05; Figure 4). In the control group, all analyzed HRV parameters also remained constant throughout the study (all *p* > 0.05; Figure 4).

### 3.4. Left Atrial Hcn1, Hcn2, Hcn4, and Pitx2 RNA Expression in the Electrically Stimulated and Non-Stimulated Rats

Left atrial RNA expression of *Hcn1* and *Hcn2* was similar in the two groups (both *p* > 0.05; Table 3). However, left atrial RNA expression of *Hcn4*, encoding for the pacemaker current, *I_f_*, was significantly higher in the AF compared to the control group (*p* = 0.03). Oppositely, left atrial RNA expression of *Pitx2* was significantly lower in the AF compared to the control rats (*p* = 0.02; Table 3).

## 4. Discussion

The main findings of the present study are that: (1) transesophageal atrial electrical stimulation induces AF in rats in the absence of any adjuvant factors; (2) long-term atrial electrical stimulation leads to spontaneous episodes of AF, the number of which increases progressively with longer duration of atrial stimulation; (3) in the stimulated rats, there is a progressively slower HR and increased SDNN, suggesting a relative increase in cardiac parasympathetic modulation; and (4) all these changes are accompanied by an increase in left atrial RNA expression of *Hcn4*, encoding for *I_f_*, and a decrease in left atrial RNA expression of *Pitx2*.

### 4.1. Long-Term Atrial Burst Pacing Induces Spontaneous Atrial Fibrillation in Rats

The association of AF with increased morbidity and mortality leads to numerous problems in clinical practice, especially since its prevalence is continually increasing [1]. Clarification of the mechanisms involved in AF occurrence and maintenance and the identification of new therapeutic targets largely depend on a detailed evaluation of clinically relevant experimental animal AF models.

Numerous in vivo experimental models have been successful in inducing AF in large animals, such as dogs or sheep [3,4]. However, large experimental animal models have the disadvantage of increased costs, related to the need for important resources such as space, specialized medical care, and equipment [3,4]. Small animal models could provide important additional insights into the pathophysiological mechanisms underlying AF.

More than a hundred years ago, Walter Garrey noted that small hearts recover very quickly from the fibrillary state [12]. This observation was the basis for the “critical mass” hypothesis, which suggested that fibrillation rhythms cannot be maintained in small-sized hearts [13]. Nevertheless, models of AF have been developed and implemented in small animals [5,6,7,14]. However, those models traditionally used adjuvant factors to ensure AF occurrence, evaluating only the effect of acute atrial stimulation on AF occurrence, and/or requiring long follow-up [6,7].

We recently developed a new model of spontaneous AF induced by long-term programmed transesophageal atrial electrical stimulation in rats [8,9]. The initial study primarily focused on establishing the feasibility of inducing AF in rats through long-term transesophageal pacing [8,9]. While this was a crucial first step, there was a need for more comprehensive investigation of that model. An in-depth understanding of the mechanisms underlying the occurrence of spontaneous AF in that model [8,9] is crucial for its use in future experimental studies. In the current study, we first validated the AF model previously developed in our laboratory and thus confirmed that long-term atrial pacing leads to the occurrence of spontaneous AF in rats.

In our model, the long-term (10-day) application of transesophageal atrial pacing created the atrial substrate necessary for spontaneous (secondary) AF occurrence from the first week of atrial pacing, when 85.7% of the stimulated animals presented spontaneous AF. Moreover, episodes of spontaneous AF were seen in all stimulated rats, not only during, but also after the end of the stimulation protocol. Although the spontaneous occurrence of AF after the initiation of atrial pacing showed only a strong trend towards statistical significance, the physiological significance of spontaneous AF occurrence is not solely dependent on statistical significance. The progressive increase in AF episodes across multiple ECG monitoring periods supports the relevance of this AF model. Thus, the present study confirms that the “AF begets AF” concept also applies to small animals, despite their reduced atrial mass. Of the animals that developed AF, the percentage of pacing cycles followed by AF episodes varied, as did the duration of AF episodes (ranging from 31 to 279 s). This inter-individual variability suggests potential differences in atrial remodeling induced by atrial electrical stimulation.

Occurrence of rapid focal discharges has been incriminated as a major contributor to the “AF begets AF” phenomenon [3,13,14]. In a canine model of AF induced by chronic rapid atrial pacing, intermittent focal discharges were observed in the pulmonary veins [15]. In line with this concept, in the present study, atrial pacing led to a progressive increase in the number of APBs during the atrial stimulation protocol. Also, the duration of previous episodes of AF seems to dictate the susceptibility of the atrium to develop new episodes of spontaneous AF following APBs [5,14]. In a study performed in goats, after 6 h of AF, a single premature stimulus increased the vulnerability of the atrium to develop paroxysms of AF [5]. After 24 h of AF, the vulnerability of the atria to fibrillation was further increased. Moreover, after 24 h of AF, the application of extra stimuli led to 20 s of rapid, irregular atrial activity, while after 2 weeks of AF, the application of extra stimuli induced sustained episodes of rapid, irregular atrial activity that did not finish spontaneously [5]. In the present study, the increased duration of the atrial electrical stimulation protocol (10 days), together with the increased inducibility of AF, led to an increase in APB burden, increased atrial susceptibility to AF occurrence, and promoted the development of spontaneous AF.

Compared with larger animal models, such as dogs and pigs, which better reproduce human cardiac electrophysiology and spontaneous AF, small animal models of AF offer a practical approach for mechanistic studies [3]. Rodent models, including our AF model, provide a valuable tool for assessing arrhythmia mechanisms due to their cost-effectiveness and rapid reproducibility [3]. However, small animal experimental models have important limitations, including anatomical differences, high baseline heart rates, and the need for artificial induction of AF, which may limit direct translatability to human AF. Clinically, AF is influenced by many factors, including aging, hypertension, obesity, diabetes, and heart failure, all of which contribute to atrial remodeling and increased susceptibility to AF [16]. Replicating all of these factors in an experimental model of AF is difficult. Therefore, by validating a rat model of AF that mimics certain alterations observed in human AF, our study provides a useful platform for investigating disease mechanisms and testing novel therapeutic strategies in a controlled experimental setting.

### 4.2. Long-Term Atrial Burst Pacing Induces a Progressive Increase in Cardiac Vagal Modulation

The substrate for spontaneous AF occurrence following rapid atrial pacing has been linked to a process of atrial remodeling induced by the rapid heart rates [5]. Structural atrial changes, alterations in atrial ion channel expression, and modifications of the cardiac autonomic nervous system have been shown to occur in this setting [17,18]. Previous studies described an increase in atrial sympathetic innervation and activity following rapid atrial pacing [14]. In the present study, LF, a marker of sympathetic activity, was not affected by long-term atrial pacing. Similarly, none of the parasympathetic markers (i.e., SDNN, RMSSD, pNN5, HF) was affected by atrial pacing, and there was no apparent sympatho-vagal imbalance in the electrically stimulated compared to the non-stimulated rats, as reflected by the similar LF/HF ratio in the two groups. However, in the arrhythmic rats, SDNN increased progressively after the initiation of the atrial electrical stimulation protocol, a change that was not seen in the control rats. In line with this finding, the mean 24 h heart rate also decreased progressively in the stimulated rats after the initiation of the atrial electrical stimulation protocol. This progressive increase in SDNN, coupled with the progressive decrease in heart rate observed in the AF group indicate a change in the sympatho-vagal balance towards increased parasympathetic modulation in response to the atrial electrical stimulation protocol. However, no similar trend was observed in other parameters associated with parasympathetic modulation and there was no significant difference between the control and AF groups regarding the HRV parameters. Future studies will thus have to clarify the exact autonomic changes that occur following prolonged atrial pacing.

**Figure 5:**
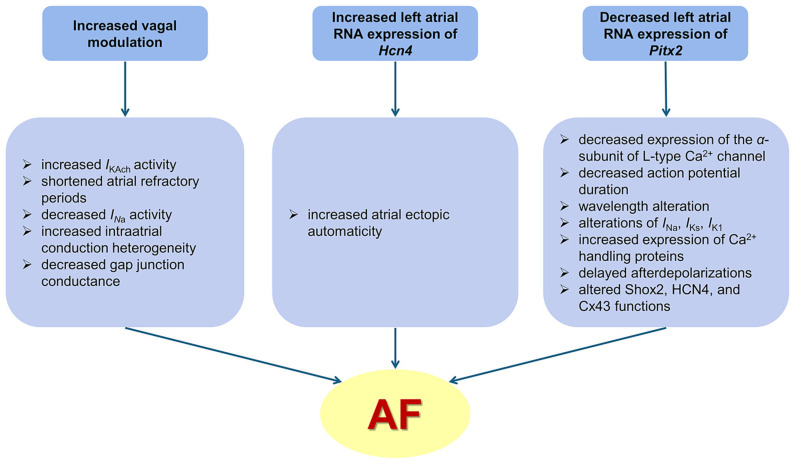
Central illustration of the mechanisms likely to be involved in the occurrence of spontaneous atrial fibrillation in the described model. AF—atrial fibrillation; Cx43—connexin 43; *I_K1_*—inwardly rectifying potassium current; *I_KAch_*—acetylcholine-activated potassium current; *I_Ks_*—slow delayed rectifier potassium current; *I_Na_*—voltage-gated sodium current; *Hcn4*—hyperpolarization-activated cyclic nucleotide-gated channel 4; *Pitx2*—paired-like homeodomain transcription factor 2; RNA—ribonucleic acid.

A comparable autonomic change, with progressive, relative vagal hypertonia, was previously described in a model of spontaneous AF in aging rats with spontaneous arterial hypertension [10]. In addition, in those rats, direct and indirect parasympathetic stimulation exhibited strong proarrhythmic effects [10]. Given the intense atrial proarrhythmic effects of vagal hyperactivity [19,20,21], this autonomic change may be at least partly responsible for the occurrence of spontaneous AF in our model. By augmenting *I_KAch_*, increased parasympathetic tone shortens the refractory periods, increases intra-atrial conduction heterogeneity, and decreases the wavelength for reentry, promoting reentry and AF (Figure 5) [14,20].

### 4.3. Long-Term Atrial Burst Pacing Induces Proarrhythmic Changes in Left Atrial Expression of Atrial Fibrillation-Related Genes

Atrial fibrillation has been associated with molecular changes leading to alterations in ion channels, signaling pathways, and structural proteins. A key role in establishing and maintaining the normal heart rhythm is played by the HCN channels, the molecular correlates of the pacemaker current (*I_f_*) [22,23]. Clinical studies have shown that, in patients undergoing corrective cardiac surgery, right atrial expression of f-channel isoforms (HCN1, HCN2, and HCN4) and *Hcn4* mRNA levels, predominantly reflecting sinus node levels, are significantly reduced in patients with AF compared to those in sinus rhythm [24]. However, HCN isoforms are also expressed in the left atrial myocardium and left atrial HCN overexpression has been linked with increased likelihood of AF [23,25,26]. By increasing *I_f_* expression, atrial *Hcn4* (the main gene encoding for *I_f_* in the sinus node) overexpression can promote cardiac ectopic automaticity, leading to AF [22,23]. Experimental studies have shown that *Hcn2* and *Hcn4* expressions were lower in the sinoatrial node, but higher in the left atrium and pulmonary veins in animals with AF compared with sinus rhythm controls [23,25,26]. Increased atrial expression of *Hcn4* was also reported in aging rats [27]. In line with these data, in the present study, rats with long-term atrial pacing-induced spontaneous AF presented increased *Hcn4* expression in the left atrium. Given their central role in atrial arrhythmogenicity, these changes are likely to have contributed to the spontaneous occurrence of AF in our model (Figure 5).

The concomitant left atrial *Pitx2* downregulation is likely to have further promoted the increased propensity to spontaneous AF seen in this model. Similar changes have often been reported in both patients and animals with spontaneous AF. Studies performed on left atrial samples from patients with AF undergoing thoracoscopic AF ablation showed decreased mRNA levels of *Pitx2* [28,29]. Altered left atrial levels of *Pitx2*-dependent miRNAs were associated with AF in patients with valvular heart disease [30] and left atrial cardiomyocyte *Pitx2* expression predicted AF recurrence after AF ablation [31]. In aging spontaneously hypertensive rats with spontaneous AF, left atrial *Pitx2* downregulation was also observed [32]. In that model, *Pitx2* downregulation was attributed to gene hypermethylation, and administration of the demethylating agent decitabine significantly reduced the AF burden [33]. In addition, rapid atrial pacing has been shown to induce atrial tachyarrhythmias in Pitx2(null+/−) adult mice [29]. In the atria, *Pitx2* plays the role of a transcriptional regulator, and its decrease has been shown to induce proarrhythmic cellular and molecular changes [34,35,36]. In Pitx2c+/− mice, action potential duration was reduced, affecting wavelength and AF inducibility [37]. In NppaCre + Pitx2−/− and Pitx2c+/− mice, atrial repolarization and resting membrane potential were affected by the altered expression of several potassium channels [35,37]. In *Pitx2*-deficient mice, Chinchilla et al. demonstrated a decrease in *I_K1_* channel expression [35]. Alterations in *I_Ks_*, *I_Na_*, and *I_Ca, L_* associated with *Pitx2* expression have also been reported [31,38]. Additionally, PITX2 controls calcium handling via Wnt signaling, as well as Shox2, HCN4, and Cx43 functions, all of which participate in AF-related electrical remodeling [39,40,41,42]. In addition to its effects on atrial electrophysiology, PITX2 misexpression also contributes to proarrhythmic atrial structural changes, which further increase AF susceptibility. PITX2 directly regulates the genes that stabilize intercalated discs in the postnatal atrium [34]. In Pitx2c+/− mice, Bmp10, a PITX2-repressed atrial protein, is highly upregulated, suggesting that PITX2 may also control the atrial chambers’ dimensions [31]. In line with those data, our rats with spontaneous AF following long-term atrial pacing also presented decreased left atrial RNA *Pitx2* expression, which is likely to have contributed to pacing-induced proarrhythmic atrial remodeling and to the occurrence of spontaneous AF in this model (Figure 5).

### 4.4. Translational Perspective

The present study confirms the ability of long-term transesophageal atrial pacing to induce spontaneous AF in rats and validates this as a successful animal AF model that can be used to further clarify AF pathophysiology and identify and test novel therapeutic approaches. In addition, the current study provides important insights into the proarrhythmic autonomic and molecular changes that occur in this model. The presence of relatively increased vagal modulation, increased left atrial *Hcn4* and decreased left atrial *Pitx2* expression, previously reported in AF patients, further validate this model as a clinically relevant animal AF model.

### 4.5. Study Limitations

Our study has a number of limitations. The sample size, particularly of the control group, was relatively small and this may have reduced the power of our statistical analyses. However, the sample size was chosen based on observations from previous studies and ethical considerations.

The short, self-limiting, irregular atrial tachyarrhythmia episodes observed in our model do not meet the traditional 30 s duration commonly used to define clinical AF, nor do they progress to persistent or permanent forms within the time frame of our study. However, this is a normal feature for small rodents, such as rats [12,13]. Long-term, persistent episodes of AF (i.e., reentry) require a sufficiently large tissue mass. Given the very small size of the rat atria, long-term maintenance of reentry circuits is essentially impossible. Despite this limitation, our model introduces a novel approach to studying AF in rats, contributing to the broader understanding of arrhythmia mechanisms in smaller, non-traditional animal models.

Autonomic changes were evaluated using only HRV analysis. Muscarinic receptor blockade or vagal nerve activity recordings could bring additional information regarding the influence of the parasympathetic nervous system on the occurrence of spontaneous AF in this model. *Hcn4* and *Pitx2* changes were only assessed by RNA quantification. Although altered gene expression is expected to result in abnormal protein variations, other post-transcriptional or post-translational regulatory mechanisms may influence protein level and function. Future studies should incorporate Western blot analysis to validate our findings and provide a more comprehensive understanding of the role of HCN channels and *Pitx2* in atrial remodeling and AF pathogenesis. To confirm the impact of *Hcn4* upregulation, the evaluation of HCN proteins and *I_f_* activity in the left atrium would be of interest. Evaluation of the presence and severity of atrial structural remodeling in this model would also be of interest.

## 5. Conclusions

In this study, we successfully validated and characterized a novel model of increased atrial arrhythmogenicity and AF induced by long-term transesophageal programmed atrial electrical stimulation in the rat. Autonomic remodeling, characterized by a relatively increased parasympathetic input, and left atrial molecular remodeling, characterized by the increased expression of *Hcn4* and decreased expression of *Pitx2*, are likely to play central roles in AF pathogenesis in this model. Further research is needed to explore the full spectrum of atrial proarrhythmic changes that occur in this model and its potential implications for the development of novel antiarrhythmic strategies.

## Figures and Tables

**Table 1:** Average heart rate of electrically stimulated (AF) and non-stimulated (control) rats during the four periods of continuous ECG monitoring.

ECG Monitoring Period	AF Group (n = 7)	Control Group (n = 5)	*p* Value
Before stimulation	344.7 ± 9.3	329.4 ± 3.8	0.17
Week 1	330.6 ± 4.9	320.8 ± 12.5	0.50
Week 2	320.4 ± 5.1	315.9 ± 10.1	0.70
After stimulation	318.0 ± 4.4	312.2 ± 12.4	0.60

AF—atrial fibrillation; week 1—at the end of the first week of atrial pacing; week 2—at the end of the second week of atrial pacing. Data are expressed as means ± standard error of the mean; *p*-values were obtained using the Welch corrected unpaired *t*-test.

**Table 2:** Heart rate variability parameters in the electrically stimulated (AF) and non-stimulated (control) rats during the four periods of continuous ECG monitoring.

Parameter	ECG Monitoring Period	AF Group (n = 7)	Control Group (n = 5)	*p* Value
Time domain				
SDNN (s)	Before stimulation	20.69 [19.05–22.48]	21.47 [18.75–27.46]	0.46
	Week 1	21.37 [21.10–22.12]	23.34 [20.71–25.13]	0.53
	Week 2	23.11 [20.06–23.40]	21.52 [10.03–24.88]	0.82
	After stimulation	26.07 [23.97–28.45]	28.07 [20.52–28.94]	0.53
RMSSD (s)	Before stimulation	4.54 [3.55–5.11]	3.93 [3.65–4.87]	0.62
	Week 1	4.12 [3.54–4.91]	4.18 [4.61–5.97]	0.20
	Week 2	4.30 [3.29–4.77]	4.13 [3.44–5.05]	0.76
	After stimulation	4.22 [3.42–4.71]	3.81 [3.37–4.57]	0.65
pNN5 (%)	Before stimulation	18.50 [13.08–22.29]	17.38 [13.80–23.58]	0.93
	Week 1	17.30 [12.90–22.33]	18.91 [16.86–22.50]	0.42
	Week 2	17.42 [11.47–23.83]	16.60 [13.08–19.75]	0.87
	After stimulation	17.35 [10.46–21.75]	14.82 [13.37–20.48]	0.68
Frequency domain				
LF (ms^2^)	Before stimulation	2.11 [2.02–2.46]	2.13 [1.68–2.52]	0.82
	Week 1	2.27 [1.80–2.37]	2.46 [1.48–2.99]	0.86
	Week 2	2.58 [2.05–2.90]	2.40 [1.57–2.98]	0.61
	After stimulation	2.58 [1.71–2.81]	2.58 [2.23–2.76]	0.42
HF (ms^2^)	Before stimulation	8.58 [6.53–9.46]	7.52 [6.61–7.83]	0.22
	Week 1	6.17 [4.38–7.53]	7.33 [6.50–8.92]	0.27
	Week 2	5.86 [3.29–9.14]	6.17 [4.70–7.42]	0.83
	After stimulation	5.84 [3.27–7.79]	5.64 [3.99–7.49]	0.66
LF/HF	Before stimulation	0.28 [0.18–0.31]	0.35 [0.235–0.36]	0.23
	Week 1	0.29 [0.27–0.30]	0.21 [0.17–0.35]	0.59
	Week 2	0.27 [0.25–0.31]	0.30 [0.23–0.36]	0.52
	After stimulation	0.22 [0.18–0.25]	0.26 [0.22–0.32]	0.19

AF—atrial fibrillation; HF—high-frequency (0.6–2.5 Hz) signals; LF—low-frequency (0.3–0.6 Hz) signals; pNN5—percentage of adjacent RR intervals that differed by >5 ms; RMSSD—root-mean-square of successive RR-interval differences; SDNN—standard deviation of normal RR intervals. Data are expressed as medians and interquartile range; *p*-values were obtained using the Mann–Whitney U test.

**Table 3:** Left atrial RNA expression of four atrial fibrillation-related genes in electrically stimulated (AF) and non-stimulated (control) rats.

AF-Related Gene	AF Group (n = 7)	Control Group (n = 5)	*p* Value
*Hcn1*	1.42 ± 0.09	1.48 ± 0.11	0.76
*Hcn2*	1.39 ± 0.03	1.33 ± 0.01	0.14
*Hcn4*	1.42 ± 0.02	1.35 ± 0.01	0.03
*Pitx2*	1.22 ± 0.04	1.40 ± 0.04	0.02

AF—atrial fibrillation; *Hcn*—hyperpolarization-activated cyclic nucleotide-gated channels; *Pitx2*—paired-like homeodomain transcription factor 2. The values were obtained by normalizing the expression levels of the target genes with that of the glyceraldehyde 3-phosphate dehydrogenase housekeeping gene. Data are expressed as mean ± standard error of mean; *p*-values were obtained using the Welch corrected unpaired *t*-test.

## Data Availability

The datasets can be obtained from the corresponding author on request due to laboratory policy.

## Abbreviations

The following abbreviations are used in this manuscript:

AF	atrial fibrillation
ABPs	atrial premature beats
Cx43	connexin 43
HCN	hyperpolarization-activated cyclic nucleotide-gated channels
IK1	inwardly rectifying potassium current
IKAch	acetylcholine-activated potassium current
IKs	slow delayed rectifier potassium current
INa	voltage-gated sodium current
LF	low-frequency components
PITX2	paired-like homeodomain transcription factor 2
pNN5	proportion of adjacent RR intervals that differed by >5 ms
RMSSD	root mean square of successive RR-interval differences
SDNN	standard deviation of normal RR intervals
